# Strengthening of Mg-6Al-1Zn Alloy via Simultaneous Loading and Aging

**DOI:** 10.3390/ma15082782

**Published:** 2022-04-10

**Authors:** Jiejun He, Lushu Wu

**Affiliations:** 1 School of Materials and Energy Engineering, Guizhou Institute of Technology, Guiyang 550003, China; 2 School of Mechanical Engineering, Guizhou Institute of Technology, Guiyang 550003, China; wulushu@git.edu.cn

**Keywords:** magnesium alloys, aging, precipitate, strengthening

## Abstract

An obvious strengthening phenomenon has been observed in the Mg-6Al-1Zn (AZ61) alloy after simultaneous loading and aging at 170 °C. Being different to aging after pre-strain, the simultaneous loading and aging can obviously increase the yield stress of the alloy. Microstructural analysis shows that a larger quantity of the Al_12_Mg_17_ can be obtained by simultaneous loading and aging in a relatively short aging time, compared with aging after pre-strain. It is speculated that the loading during aging is more beneficial for nucleation of the precipitates. In the same aging time, it is found that the sample subjected to simultaneous loading and aging shows a higher yield stress than the sample aged after pre-strain. To extend aging time, a large quantity of Al_12_Mg_17_ can be obtained in the pre-strained sample. However, it is demonstrated that the yield stress of the sample subjected to aging after pre-strain is lower than that of the sample subjected to simultaneous loading and aging, despite these two samples containing the same quantity of precipitates. It is speculated that the occurrence of the precipitates plays a role in preventing dislocation gliding and twin expanding, thus leading to a strengthening effect. Additionally, atoms segregated in twin boundaries may partly strengthen the material. It is found that a large quantity of precipitates can be obtained in a relatively short aging time by using the simultaneous loading and aging, reducing the softening effect caused by aging. The observed phenomenon may provide a new strategy for strengthening magnesium alloys.

## 1. Introduction

In recent years, magnesium and its alloys have attracted great attention in energy saving because of their low weight [1,2,3]. However, the wider application of these alloys is restricted due to their poor formability at room temperature and relatively low yield strength in contrast to steels and aluminum alloys. Therefore, to improve the mechanical properties, especially the yield stress and the elongation of magnesium alloys are of general interest for broadening the application of magnesium and its alloys [4,5,6,7]. Many techniques have been developed to enhance the properties of magnesium alloys, containing alloying, severe plastic deformation, aging strengthening, and so on. Recently the process of pre-deformation and subsequent annealing attracts many researchers because it has a strengthening effect on magnesium and its alloys if the annealing temperature is suitable. For instance, Nie et al. [8] reported a strengthening phenomenon in pre-deformed Mg-Gd alloys during subsequent annealing because the equilibrium segregation of solute atoms into twin boundaries provided a pinning effect, leading to an annealing hardening effect. The same strengthening phenomenon was also found in magnesium alloy AZ31 [9]. Strengthening effect caused by the pinning of basal dislocation by G.P. zones generated during annealing was also found in a dilute Mg-Zn-Ca alloy [10]. It is surprising that this strengthening phenomenon is also found in pure magnesium [11]. The rearrangement of the dislocation structure activated by annealing processes and the blocking of dislocations were responsible for that strengthening phenomenon. In addition to these, the orientation change caused by pre-deformation and subsequent annealing under relatively high temperature [12], grain refinement by recrystallization after cold plastic deformation [13] and precipitates generated during annealing after deformation [14,15,16] all affect the mechanical behavior of magnesium alloys. Among all the possible strengthening mechanisms for annealing hardening, the occurrence of precipitates to prevent dislocation gliding and twin expanding, thus leading to a strengthening effect, is important for magnesium alloys. It is reported that pre-deformation has an obvious influence in precipitation behavior in magnesium alloys. Our previous work [14] had found that there were a few precipitates appearing in the AZ61 alloy during annealing if the alloy was not subjected to pre-deformation. However, if the alloy was subjected to pre-deformation and then annealed under a relatively low temperature, a higher quantity of second-phase particles, for instance, Al_12_Mg_17_, would appear in the alloy. It is speculated that the existence of inner stress has obvious effects on the precipitation behavior during subsequent annealing.

Although the pre-deformation, such as pre-compression, pre-tension and pre-rolling, has an important effect on precipitation behavior during subsequent annealing, the pre-strained alloy still may be softened if the annealing time is long, despite a large mumble of fine precipitates being obtained during the annealing and the grain size having no obvious change [15,16]. That means, precipitation hardening and recovery softening may take place simultaneously in the alloy during annealing and the final strengthening effect depends on the comprehensive role of these two effects. If a high quantity of precipitates can be obtained in a relatively short annealing time, the softening effect produced by recovery may be reduced, thus leading to a higher strengthening effect. In the previous work, pre-strain and annealing were usually used in different steps. Here, we report a method of obtaining a high quantity of precipitates in a relatively short aging time by process of simultaneous load and aging. It may provide a new viewpoint for strengthening magnesium alloys.

## 2. Material and Experimental Procedures

### 2.1. Loading and Aging Procedure

**Figure 1:**
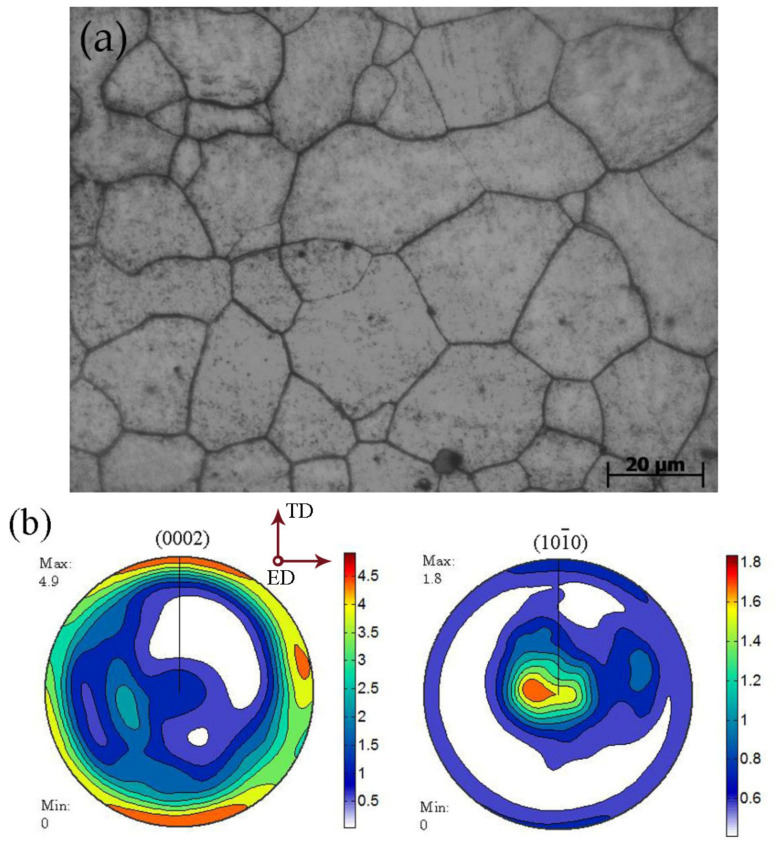
(**a**) The optical microstructure and (**b**) The {0002} and {10$\overset{-}{1}$0} pole figures of the as-received material used in this study.

The starting material used in this study was a commercially purchased AZ61 rod. The compositions of the AZ61 alloy (mass %) was given in Table 1. Microstructural analysis revealed that the obtained AZ61 alloy was twin-free and it exhibited nearly equiaxed grains with a mean grain size of ~20 μm, as indicating in Figure 1a. As shown in Figure 1b, there was a typical hot-extruded texture in the magnesium alloy and it is beneficial for generating tensile twins if a compressive stress was applied along the extruded direction (ED).

**Figure 2:**
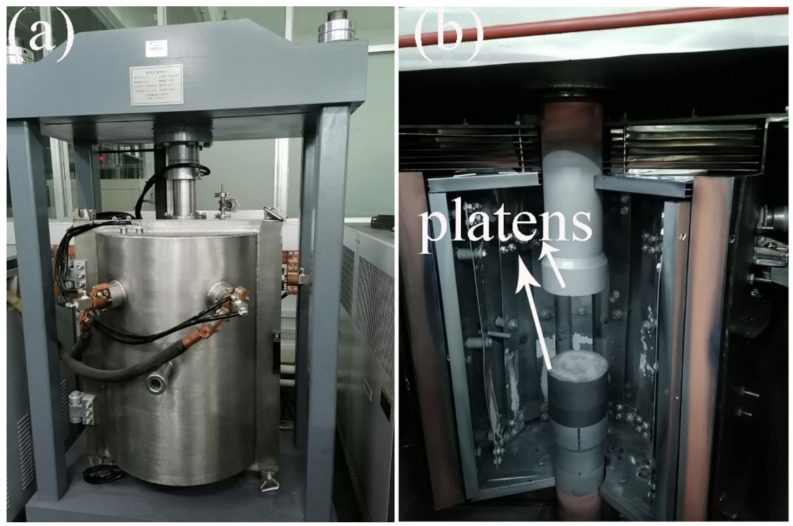
The image of the diffusion welding furnace with a loading system used in this study. (**a**) heating furnace; (**b**) loading system.

Simultaneous loading and aging (signed as one-step process) was performed in a diffusion welding furnace with a loading system (Figure 2). A metallic indenter was used for loading. Samples (8 mm diameter and 12 mm height) were machined from the extruded rod along ED by mean of wire-electrode cutting. The maximum load was chosen to be 8800 N and it could be maintained during the aging progress. When the load reached the maximum value, the applied stress was estimated to be about 175 MPa. The aging temperature in this study was chose to be 170 °C. According to previous reports [9,16], the grain size, twin structure and texture can be well maintained under the selected temperature or a slightly high one. That means, the texture evolution, grain growth and change of twin structure were unapparent and the effects of these factors on subsequent deformation can be ignored. Additionally, aging after pre-strain (signed as two-step process) was conducted in the same system to investigate the possible effects of these processes on microstructure and mechanical behavior of the alloy.

### 2.2. Microstructural Characterization and Mechanical Test

Microstructural observation was carried out by optical microscopy (OM, Shunyu LTD, Ningbo, China) and scanning electron microscopy (SEM, Nova Nano SEM 450, FEI LTD, Hillsboro, OR, USA) using an accelerating voltage of 20 kV after careful polishing and etching with an acetic picral solution containing 5 mL acetic acid, 6 g picric acid, 10 mL H_2_O and 100 mL ethanol. The pole figures in this study were measured using a Rigaku D/max-2500 X-ray diffraction (XRD, Rigaku, Tokyo, Japan) to observe the cross section, using Cu k_α_ radiation (Wavelength λ = 0.15406 nm) at 45 kv and 150 mA with a sample tilt angle ranging from 0~80°. Additionally, 0 XRD patterns were obtained through the same machine at 40 kv and 150 mA for identifying the precipitation phase. Electron backscattered diffraction (EBSD, TSL, Tennessee, TN, USA) analysis was carried to identify the twins on FIE Nova SEM 450 scanning electron microscope equipped with an TSL OIM-EBSD system using a step size of 0.5 μm. Sample for EBSD was ground mechanically followed by electrochemical polishing in commercial AC2 solution. The TSL OIM analysis software (TSL, Tennessee, TN, USA) was utilized to process the data obtained from the electron backscattered diffraction. Transmission electron microscopy (TEM, Carl Zeiss Libra-200, Zeiss, Oberkochen, Germany) was used for further studies of microstructure. Thin foil TEM samples were prepared using mechanical polishing and ion-shinning. The TEM observations and energy dispersive X-ray spectrometry (EDS) analysis were carried out on a Carl Zeiss Libra-200 equipped with an EDS detector and operated at 200 kV.

Finally, all the aged samples were reloaded to failure at room temperature to investigate the effects of these processes on mechanical behavior using a CMT5105 material test machine (MTS LTD, Shenzhen, China) at a constant rate of 10^−3^ s^−1^.

## 3. Results

### 3.1. Effects of Simultaneous Loading and Aging on Microstructure

**Figure 3:**
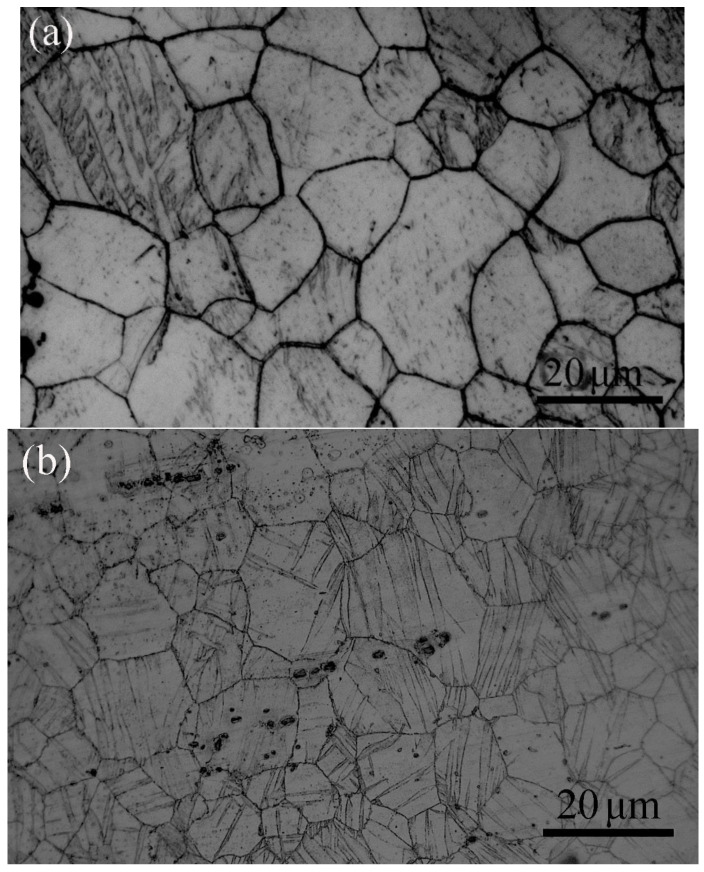
(**a**) The microstructure of the AZ61 alloy subjected to a compressive stress of 155 MPa and (**b**) the microstructure of sample subjected to a compressive stress of 175 MPa.

**Figure 4:**
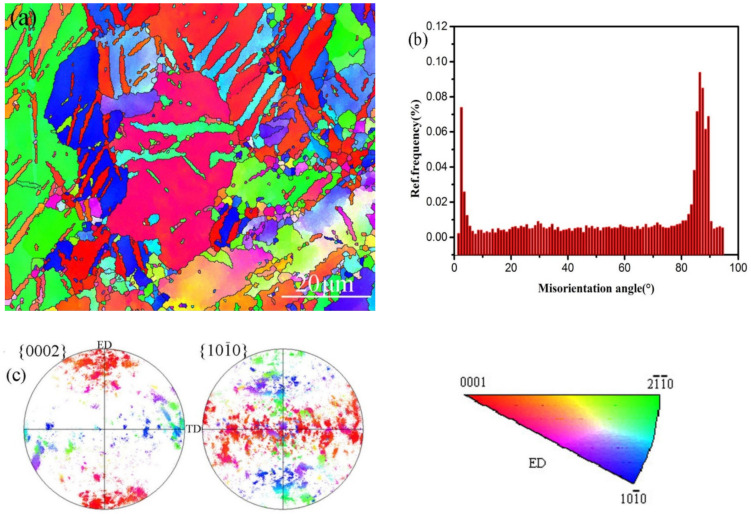
EBSD results illustrating the occurrence of {10$\overset{-}{1}$2} twins:(**a**) Inverse pole figure map of the sample subjected to 175 MPa, (**b**) Misorientation angle showing the appearance of 86.3 ± 5° and (**c**) pole figure showing basal planes parallel to ED after strain. ED stands for the extrusion direction and TD stands for the transverse direction of the extruded bar.

The applied stress in this study varied from 0 MPa to 175 MPa and the maximum stress could be maintained during aging. Figure 3a,b illustrate the microstructures of the AZ61 alloy subjected to a compressive stress of 155 MPa and that subjected to a compressive stress of 175 MPa, respectively. It can be found in Figure 3a that a few twins appear in the alloy when the applied stress was 155 MPa. However, a great deal of lenticular shaped twins appeared in the sample when it subjected to 175 MPa. It is well known in magnesium alloy that the tensile twins will generate if there is compressive stress perpendicular to *c* axis or tensile stress parallel to *c* axis. As shown in Figure 4a, twins can be observed in the sample subjected to 175 MPa. The EBSD results highlighted the misorientation of ~86.3 ± 5°, revealing that these twins were {10$\overset{-}{1}$2} type twins, as shown in Figure 4b. It was found that most of the {0002} planes of the grains were parallel to ED in the obtained alloy, as indicating in Figure 1b. However, many {0002} planes of the grains became to be perpendicular to ED because of the occurrence of {10$\overset{-}{1}$2} twins, leading to a transformation of ~86.3° in orientation in the twinned area, as shown in Figure 4c.

**Figure 5:**
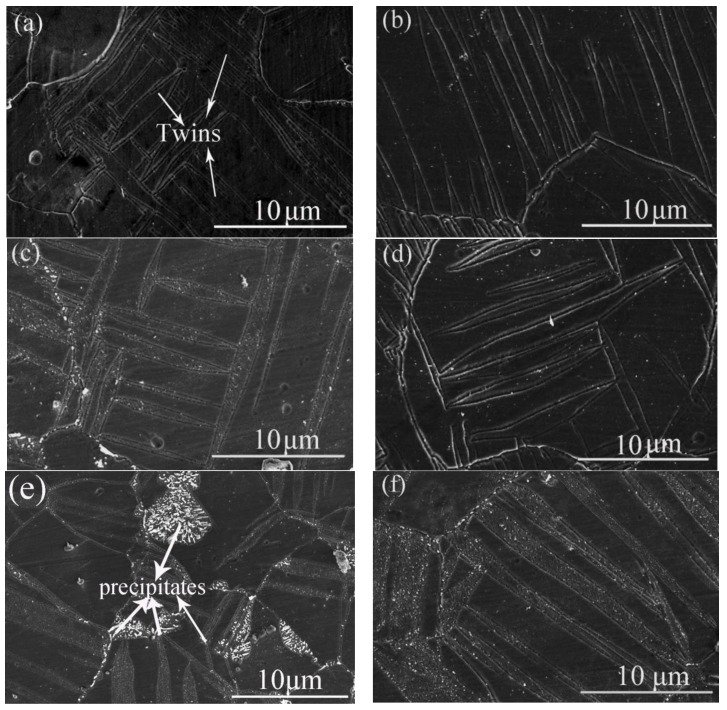
SEM micrographs demonstrating the microstructure of (**a**) the sample subjected to 175 MPa, (**b**) the sample subjected to 175 MPa and then aged at 170 °C for 1 h, (**c**) the sample subjected to simultaneous loading (175 MPa) and aging at 170 °C for 1 h, (**d**) the sample subjected to 175 MPa and then aged at 170 °C for 3 h, (**e**) the sample subjected to simultaneous loading (175 MPa) and aging at 170 °C for 3 h and (**f**) the sample subjected to 175 MPa and then aged at 170 °C for 10 h.

**Figure 6:**
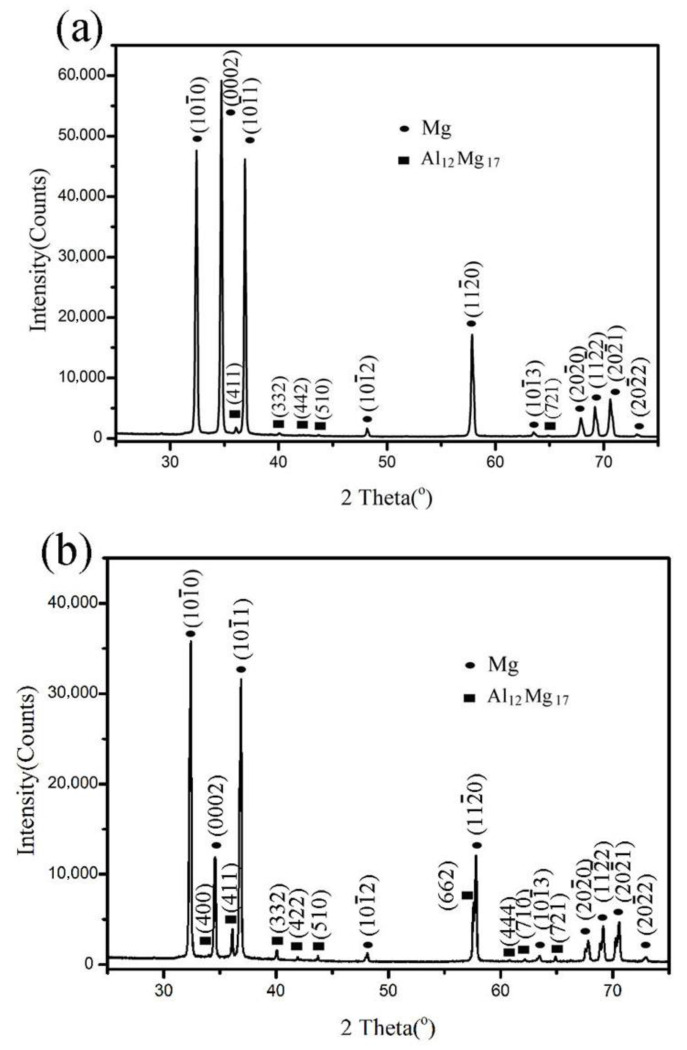
XRD patterns of (**a**) the sample subjected to 175 MPa and subsequent aging at 170 °C for 3 h and (**b**) the sample subjected to simultaneous loading and aging under the same condition.

Precipitation behaviors under different loading and aging conditions were shown in Figure 5. In the sample subjected to 175 MPa but without aging, it can be seen from Figure 5a that the amount of precipitates is very low. If the alloy AZ61 subjected to 175 MPa at first and then aged at 170 °C for 1 h, i.e., the sample was subjected a two-step process, as illustrating in Figure 5b, few second-phase particles appeared in the sample. That means the pre-strain and then aging at 170 °C for 1 h has no obvious effect on microstructure. However, if the sample subjected to simultaneous loading (175 MPa) and aging at 170 °C for 1 h, i.e., the sample was subjected to the one-step process for 1 h, the precipitation behavior was very different from that of the sample subjected to the two-step process. It can be found in Figure 5c that obvious precipitation of the second-phase particles occurred in the alloy. Many precipitates appeared in grain boundaries, twin boundaries, and within twins. If the sample subjected to the same prestress and subsequent aging at 170 °C for 3 h, a few precipitates appeared in the alloy, as shown in Figure 5d. However, a higher quantity of precipitates was obtained when the sample was subjected to simultaneous loading and aging under the same condition, as illustrated in Figure 5e. Using XRD technology, the second-phase particles were approved to be Al_12_Mg_17_, as shown in Figure 6. It was also found from the XRD results that the patterns of the Al_12_Mg_17_ of the sample subjected to the two-step process was weaker than those of the sample subjected to the one-step process, also illustrating a higher quantity of the Al_12_Mg_17_ in the one-step-processed sample than that in the two-step-processed sample.

In the sample subjected to the two-step process, however, a high quantity of precipitates could be obtained if aging time was extended. As can be seen in Figure 5f, in the sample subjected to 175 MPa and then aged at 170 °C for 10 h, when the aging time was extended to 10 h, many precipitates appeared in the alloy. It can be found that the amount of precipitates in this ample was nearly equal to that in the sample subjected to simultaneous loading and aging at 170 °C for 3 h.

### 3.2. Effects of Simultaneous Loading and Aging on Mechanical Behavior during Subsequent Deformation

**Figure 7:**
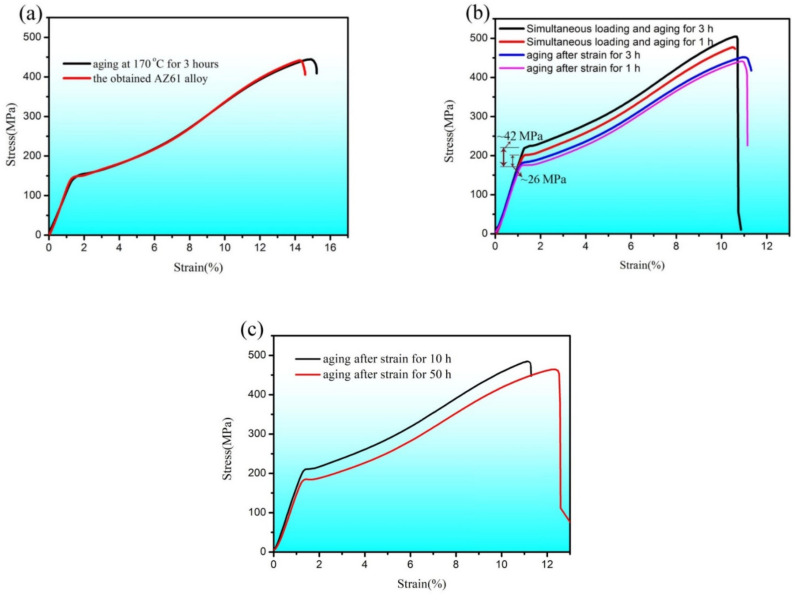
The stress–strain curves of the as-received AZ61 alloy and samples subjected to different heating and loading conditions. (**a**) the as-received alloy with and without aging; (**b**) samples subjected to aging after strain for 1 h and 3 h and simultaneous loading and aging for 1 h and 3 h respectively; (**c**) samples subjected to aging after strain for 10 h and 50 h.

In order to investigate the possible effect of aging on the mechanical properties of the obtained alloy AZ61, a sample without pre-strain was aged directly at first for 3 h and then was compressed to failure. Additionally, a sample without pre-strain and aging was compressed to failure directly. The stress–strain curves of these two samples are shown in Figure 7a. It can be found that the two curves are almost identical, illustrating the two samples show nearly equal yield stress and ultimate compressive strength. The aged sample showed a little higher elongation than that without aging. The stress–strain curves of samples subjected to different processes are given in Figure 7b. When the alloy was subjected to a stress of 175 MPa and then aging at 170 °C for 1 h, the yield stress was ~175 MPa during subsequent deformation at room temperature. However, if the alloy was subjected to simultaneous loading (175 MPa) and aging at 170 °C for 1 h, the yield stress became ~201 MPa during subsequent deformation at room temperature. It was ~26 MPa higher than that of the sample subjected to pre-strain and then aging. When a stress of 175 MPa was applied to the alloy and then aged at 170 °C for 3 h, the yield stress was nearly equal to that of the sample subjected to 175 MPa and then aged at 170 °C for 1 h, illustrating no obvious effect of this process on mechanical behavior. However, when the alloy was subjected to simultaneous loading and aging at 170 °C for 3 h, the yield stress became ~217 MPa during subsequent deformation at room temperature, which was ~42 MPa higher than that of the sample subjected to the two-step process. In the sample subjected to pre-strain and then aging, if the aging time was extended to 10 h, the yield stress was ~206 MPa during subsequent deformation at room temperature. However, the yield stress was just ~181 MPa if aging time was extended to 50 h. The yield stress as a function of process is summarized in Table 2.

## 4. Discussion

**Figure 8:**
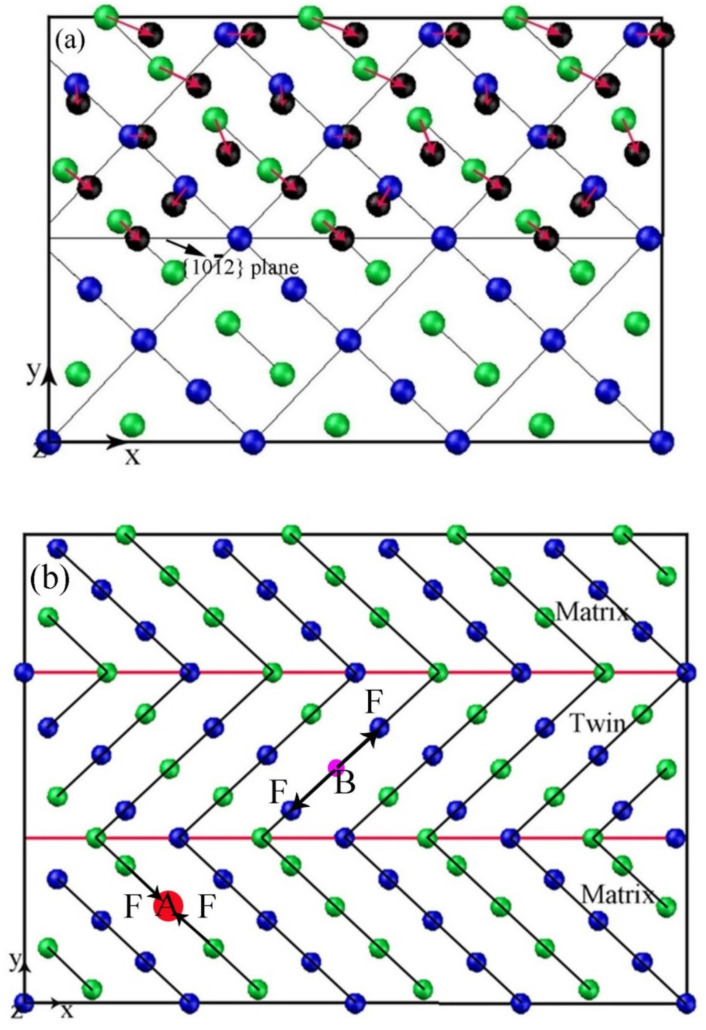
(**a**) Schematic drawing showing atomic motion in the twinning area, (**b**) Schematic drawing illustrating the relation between Mg and solute atoms.

**Figure 9:**
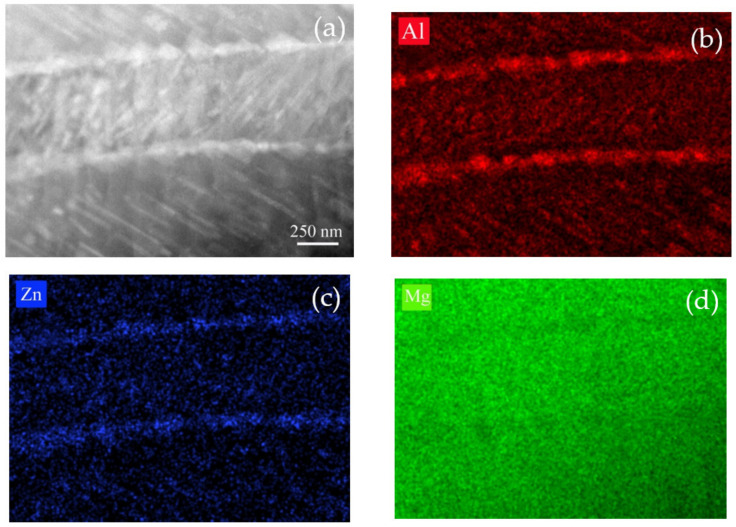
(**a**) TEM picture taken in the sample subjected to 175 MPa and then aged at 170 °C for 3 h, (**b**–**d**) Corresponding EDS maps highlighting the Al, Zn and Mg elements.

**Figure 10:**
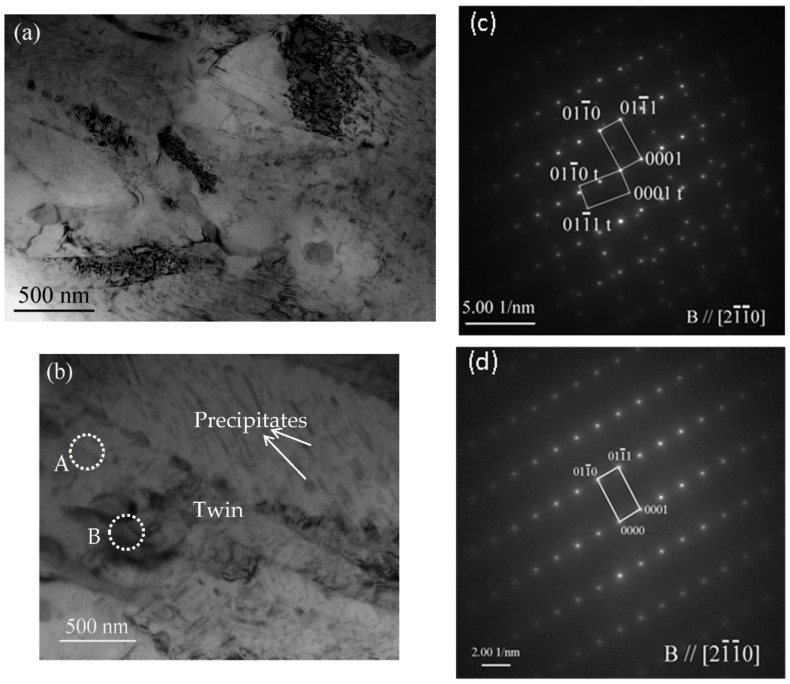
(**a**,**b**) illustrate the TEM bright field images of the sample subjected to 175 MPa and then aged at 170 °C for 1 h and the sample subjected to simultaneous loading and aging under the same condition, respectively; (**c**,**d**) show the corresponding SAED patterns of point A and B, respectively.

A high quantity of precipitates could be obtained via simultaneous loading and aging in a relatively short aging time, thus leading to an obvious strengthening effect. The effects of pre-strain and annealing on subsequent deformation behavior has been investigated by many researchers. However, these two processes were used in different steps in nearly all these investigations. It has been proved that the pre-strain had an important effect on annealing hardening behavior during the subsequent heating process. The effect factors for annealing hardening often contain transformation of dislocations, formation of GP zones, generation of the second-phase particles, and so on. Obviously, the occurrence of pre-strain has important effects on these possible hardening factors during annealing. In magnesium alloys, it is found that the existence of pre-strain is beneficial for precipitation of the second-phase particles. If a sample without pre-strain is annealed at 170 °C or a slightly higher temperature, no obvious strengthening effect is observed [14,15,16]. It is deduced that the inner stress induced by pre-strain may have effects on precipitation of the second-phase particles. In magnesium alloys, the solute atoms, such as Al and Zn, often occupy the Mg sites because their atomic sizes are similar. Twinning can be accomplished by shearing and shuffling of the atoms of the matrix [17], as indicated in the schematic drawing shown in Figure 8a. For every twin in the alloy, there are two twin boundaries in the crystal structure, as shown by the red lines in Figure 8b. As indicated by point A of Figure 8b, if there is a big solute atom whose atomic size is bigger than that of Mg, the repulsion between Mg and this solute atom will increase if there is an external pressure. This solute atom will be repelled if there is a vacancy around it. It is well known that atomic arrangement in grain boundaries is irregular [18]. Additionally, {10$\overset{-}{1}$2} twin boundaries are not strictly coherent [19]. Therefore, the solute atoms may distribute preferentially in grain boundaries and twin boundaries and form a second-phase particle. The microstructures shown in Figure 5 have indicated this trend in grain boundaries. The STEM and EDS mapping results also illustrate this trend in twin boundaries, as shown in Figure 9. If the pressure is big enough, it can be deduced that the second-phase particles may nucleate in a relatively short aging time. This trend also been proved by Zhang et al. [20] in the Mg-6Gd-1Ca alloy. If the atomic size of solute atoms, such as Al and Zn, is smaller than that of Mg, as illustrated by point B of Figure 8b, there is an attraction between the alloying and Mg atoms. If the crystal is subjected to a compressive stress, the distance between the alloying atom and Mg are reduced; thus, the attraction between these atoms declines. This indicates that the solute atoms are more active during simultaneous load and aging and leads to an enhanced precipitation during the one-step process. A further investigation on microstructures was performed using TEM technology. When the sample was subjected to 175 MPa and then aged at 170 °C for 1 h, few precipitates can be found in the alloy, as indicated in Figure 10a. However, when the sample was subjected to simultaneous loading and aging under the same stress, temperature and aging time, a great deal of precipitates appeared in the alloy, as shown in Figure 10b. The selected area electron diffraction (SAED) results shown in Figure 10c,d indicate the relationship between twin and matrix in the sample. The second-phase particles appearing in the alloy play a role of preventing dislocation movement or blocking twin growth during the subsequent deformation, leading to an enhanced yield stress. In the sample subjected to the two-step process, the inner stress induced by deformation plays an important role in nucleation and growth of the second-phase particles. However, this stress is released during the subsequent heating process. Compared with the two-step process, the external force is holding during all the heating process and it can be inferred that the driven force for precipitation will be more obvious and persistent. Therefore, it can be found that for the same aging time, there are more precipitates in the one-step processed sample. It appears that a continuous external force can affect the precipitation of the second-phase particles. It should be pointed out that the atoms segregated in twin boundaries may also partly strengthen the alloy in this study.

Certainly, a two-step process is easier to operate in actual production because the pre-strain can be obtained by many ways such as rolling, compression, and so on, and the pre-strained material can be put in a furnace for the aging. This way would reduce the dependence on complicated devices. Additionally, if there is enough aging time, a high quantity of second-phase particles will be obtained, as indicated in Figure 5f. However, a long aging time often has two effects on mechanical behavior: one is the hardening effect caused by precipitates and the other is the softening effect caused by recovery. The final effect of heating on mechanical properties depends on comprehensive function of these two effects. Although traditional views hold that recovery has little effect on mechanical properties, such as yield stress, ultimate tensile strength, hardness, and so on, and just obviously reduces the inner stress, several investigations [15,16] still pointed out that a long aging time would have a softening effect on the material despite a large quantity of second-phase particles appearing in the material. That means if the softening effect is more obvious than the hardening effect, the materials will be softened despite the many precipitates generated during aging. It should be pointed out that the amount of precipitates in a certain alloy is always limited. Therefore, strengthening effects caused by the second-phase particles will also be limited. It can be found in this study that there was a decrease in yield stress if the aging time was extended to 50 h, revealing the possible softening effect caused by recovery. It can be inferred that if the aging time is too long, the softening effect will be more obvious than the strengthening effect. Additionally, the size and dispersion degree of the precipitates are very important for hardening effects in materials. If the precipitates grow too fast during aging, the hardening effect caused by precipitates will not be obvious or even disappears. In this study, it can be found that the precipitates were generally disperse but grew in some grains in the sample subjected to simultaneous loading and aging for 3 h. However, the yield stress of this sample is still better than others in this study. It can be found that a high quantity of precipitates can be obtained in a relatively short aging time by the simultaneous loading and aging, and meanwhile can reduce the softening effect induced by recovery.

## 5. Conclusions

In this study, the AZ61 alloy was subjected to pre-strain and aging, and simultaneous loading and aging, respectively. The effects of these processes on microstructure and mechanical properties were characterized. The major conclusions can be summarized as follows:

1. Being different to aging after pre-strain, a larger quantity of the Al_12_Mg_17_ precipitates can be obtained in the AZ61 alloy subjected to simultaneous loading and aging in the same aging time. The applied stress during aging is beneficial for nucleation of the second-phase particles.
2. When the AZ61 sample was subjected to 175 MPa and then aged at 170 °C for 1 h and 3 h, respectively, no obvious increase in yield stress was observed. However, the simultaneous loading and aging shows an obvious effect on yield stress during subsequent deformation at room temperature, and it was found that there was an increase of ~42 MPa in yield stress compared with the pre-strained sample without aging. The second-phase particles generated during simultaneous loading and aging plays a role of obstacle to dislocation gliding, leading a hardening effect to AZ61 alloy. In addition, atoms segregated at the twin boundaries also contribute to the partial strengthening of the alloy under study.

## Figures and Tables

**Table 1:** Compositions of AZ61 magnesium alloy (mass%) used in this study.

Element	Al	Zn	Mn	Si	Ni	Fe	Cu	Mg
mass%	5.9	1.0	0.3	0.0070	0.0006	0.004	0.003	balance

**Table 2:** Yield stress as a function of different processes in this study.

Alloy	Process	Yield Stress
1	As-received	155 MPa
2	170 °C × 3 h	155 MPa
3	175 MPa and then 170 °C × 1 h	175 MPa
4	Simultaneous loading and aging at 170 °C for 1 h	201 MPa
5	175 MPa and then 170 °C × 3 h	175 MPa
6	Simultaneous loading and aging at 170 °C for 3 h	217 MPa
7	175 MPa and then 170 °C × 10 h	206 MPa
8	175 MPa and then 170 °C × 50 h	181 MPa

## Data Availability

All the data are already provided in the main manuscript. Contact the corresponding author if further explanation is required.
